# Evaluating the Social Marketing Success Criteria in Health Promotion: A F-DEMATEL Approach

**DOI:** 10.3390/ijerph17176317

**Published:** 2020-08-31

**Authors:** Chi-Horng Liao

**Affiliations:** Department of Communication Studies, Tzu Chi University, Hualien 97004, Taiwan; lchjerry@gms.tcu.edu.tw

**Keywords:** health promotion, health-promoting lifestyle, social marketing, Tzu Chi foundation, F-DEMATEL (fuzzy decision-making trial and evaluation laboratory)

## Abstract

Health promotion campaigns are used to raise awareness about health issues with the purpose of improving health outcomes and community wellbeing. They are important for increasing community awareness of health behavior changes. In the application of health promotion, social marketing can be used to influence changes in individual behavior. Social marketing encourages the social behavioral change of the target audience. This social behavioral change refers to the behavior of a certain number of target audiences, not just individual behavior. This research identified various social marketing success criteria to improve the performance of health promotion using decision-making method. Fuzzy decision-making trial and evaluation laboratory (F-DEMATEL) is a structural causal relation method that has been verified effectual in congregating the viewpoints of professionals and thus providing information of greater reliability in various areas. F-DEMATEL method helps to identify the core problems and direction of improvement in complex systems by quantifying the degree to which criteria attributes interact with each other. This research applied F-DEMATEL to evaluate the complex interrelation success criteria of social marketing in order to effectively implement health promotion. Several effective criteria were derived from this research. These influential criteria are “Designing effective Communication message”, “Meeting the needs of beneficiaries”, “Providing more benefit than cost”, “Marketing mix elements”, “Customer orientation”, “Organizational advantage” and “Market selection”. The practitioner must consider the needs of the recipients to accomplish a successful social marketing campaign in health promotion. Moreover, the practitioner also has to design an attractive message and marketing mix strategy to communicate the benefits of the behavioral change to the target audience. Besides, the message delivered by the known organization increases the success of social marketing in promoting healthy lifestyle. This study provides important information for the non-profit organization about selecting the significant criteria to lead to the success of the campaign.

## 1. Introduction

A healthy population is part of national competitiveness and an important driving force for the country’s sustainable development. The health policies should strive to meet people’s health needs. In line with the change of people’s health needs, the trend of health policy has shifted from focusing on medical services to promoting people’s health. To promote health and prevent diseases, instead of traditional diagnosis and treatment, is the important purpose of health promotion [1]. Health promotion facilitates individuals’ increased control over their personal health. It involves an extensive range of environmental and social interventions that are intended to benefit and protect people’s health and improve quality of life [2]. Health refers to the process of achieving comfort without disease, physical, social and psychological issues, while health promotion helps people to have the ability to control health risk factors in order to improve their health [3]. Health promotion not only enhances individual abilities or skills but also requires changing social, environmental and economic conditions, thus creating a healthy culture and an environment conducive to a healthy life.

Social marketing is a process of designing, executing and controlling programs using a combination of marketing mix and marketing research to enable target audience to accept certain concepts, ideals and measures of society [4]. The difference between social marketing and traditional marketing, especially commercial marketing, is that the product or service of traditional marketing is specific, while the product of social marketing is mostly invisible and conceptual. This refers to a change in the individual’s thinking, attitude and lifestyle or behavior. The objective of traditional marketing is to expand the market, while the objective of social marketing is to promote the well-being of the society. The main purpose of these social campaigns or programs is to increase the acceptance of ideas or principles by a particular population group. The practice of social marketing strategy is to improve the overall interests of the individual or society and to promote the voluntary acceptance and change of social behavior by the target audience. The operating process of social marketing is through market segments, identify priority experimental area and formative research. The whole process of social marketing is based on customer-oriented analysis, planning, implementation and evaluation, which are rigorous, feasible and efficient [5]. Although a social marketing campaign cannot make a major changes in cognition and behavior in a short period of time, the application of a social marketing strategy and continuous practice of it may develop the significant effect. Thus, it is important in increasing community awareness of health behavior changes. In the application of health promotion, social marketing can be used to influence changes in individual behavior [6].

Social marketing is broadly employed to influence health behavior. It is effective on a population level, and health promotion practitioners can contribute to its effectiveness. The message of social marketing may aim to prevent uncertain health and to encourage healthy behavior through persuasion, education, exposure theories and the promotion of behavioral alternatives. The social marketing used to promote a healthy lifestyle develops health promotion programs that meet the needs of a target audience. These health activities reach the needs of a wide range of individuals in order to improve the organization’s ability to achieve behavioral changes related to health, making social marketing an efficient tool for promoting a healthy lifestyle [7].

This research identified various social marketing success criteria to improve the performance of health promotion using a decision-making method. These criteria are dependent and have a causal relation structure. Correspondingly, this research proposed to apply Fuzzy decision-making trial and evaluation laboratory (F-DEMATEL) method in order to show the causal relation criteria and to increase the model applicability in terms of linguistic variables combined with triangular fuzzy numbers [8]. The F-DEMATEL method converted a complex system into a well-structured causal relationship, which reduces the relationship between criteria attributes in complex systems to cause and effect groups [9]. The method also helps to identify the core problems and direction of improvement in complex systems by quantifying the degree to which criteria attributes interact with each other. Thus, this research applied the F-DEMATEL method to evaluate the complex interrelation success criteria of social marketing in order to effectively implement health promotion.

Health promotion has often used a social marketing strategy to communicate health-related information intended to prevent disease and promote a healthy lifestyle. To effectively improve the implementation of health promotion with the help of a social marketing strategy is a significant undertaking. The concept of social marketing is most often used by the non-profit organizations. In the process of implementing social marketing by non-profit organizations, the first task is to formulate an effective message for the activities to be pursued by the organization, so as to enhance the visibility of the media through the dissemination of the information strategy, thereby affecting and gaining the response of the target audience. This research will help non-profit organizations to select the effective success criteria of social marketing in health promotion.

In this research, the main intention is to evaluate the social marketing success criteria for the effective implementation of health promotion. Kotler and Lee [10] determined fourteen key success criteria and claimed that all must be accomplished in order to assure the success of such a social marketing campaign. However, every organization sometimes struggles with limited resources, especially within the non-profit organization sector. Funding and human resources are, in a number of cases, the first two concerns before implementing any health orientated activity. In this study, the first argument is clearly stated that not all criteria can be implemented due to limited resources. Besides, several scholars also argued that some criteria are more important and significantly influential than others, which will be discussed in the next section. At end of the research, the author contributes answers and clarity to the practitioners in charge of decisions as to how to select the suitable criteria that are to be implemented when there is limited access to resources.

## 2. Literature Review

### 2.1. Health Promotion

The World Health Organization (WHO) defines “health” as a state of physical, psychological and social overall health and well-being [3]. The WHO Ottawa Charter [11] also defines health promotion as the process of enabling people to strengthen their control to improve their own health, with the goal of engaging in healthy behaviors to support the public to live a healthy lifestyle, including health education, policy, the environment and the media. Therefore, health promotion is the level of prevention in the model of community health or public health, which is to increase the measures taken by the public for their own health, including promotion of healthy behavior, focus on mental health, maintain appropriate healthy leisure activities, and build a healthy living environment [12]. Health promotion encourages healthy people to engage in healthy activities that lead to healthier lives. Thus, health promotion targets an audience composed of healthy persons with healthy behaviors. This implies combining education and environmental support to enable people to take healthy actions and have a health-promoting behavior [13].

Health-promoting behavior in an individual’s healthy lifestyle can be seen as a positive lifestyle, guiding the individuals to realize that they have a high potential to engage in healthy life [14]. It is understood that health promotion related behaviors do not necessarily mean that a person has an individual healthy lifestyle. However, it is an essential factor in developing a healthy lifestyle. The definition of health-promoting lifestyle is to maximize personal quality of life and reduce behavioral susceptibility to negative health outcomes [15]. This denotes that the function of a health-promoting lifestyle not only promotes health but also enhances the quality of life. Walker et al. [16] also define a health-promoting lifestyle as a multidimensional form of voluntary behavior and cognition in order to maintain or promote health, self-realization, and self-satisfaction. In this definition, the health-promoting lifestyle behavior extends to the mental level of individual self-realization and self-satisfaction.

Health-promoting lifestyles include behaviors at four levels: self-responsibility, healthy diet, physical cognition and stress control [17]. In addition, Ardell [18] increased and placed more emphasis on human interaction with the environment, while the modifications were expanded to self-responsibility, nutritional awareness, stress treatment, physical fitness, and sensitivity to the environment. In addition to the above, other scholars added the degree of “interpersonal support” to the point of self-realization, exercise, health responsibility, nutrition, interpersonal support and pressure treatment [16]. In summary, health-promoting lifestyle is due to the increase in disease morbidity and mortality, urging people to pay attention to health. The connotation of health-promoting lifestyle includes the psychological and spiritual aspects of environment, interpersonal, self and spiritual, in addition to the generally more common aspects of a vegetarian diet, nutrition and sleep [19].

The above-mentioned health-promoting lifestyles take time to develop. Hence, how to promote the behavioral change and further to keep the changed behavior permanently is a challenge to health promotion practitioners. In order to effectively use limited resources to solve the increasingly complex health problems, the practitioners must seek new methods and strategies, and thus, social marketing becomes one of these new endeavors.

### 2.2. Social Marketing

Social marketing is the product of the theory of marketing development to a certain stage. It uses the principles and techniques developed in the field of marketing, including exchange theory, customer orientation, focus on competitors, market research and target customer behavior analysis, market segments, target market selection (audience segmentation), marketing mix, marketing planning, execution and evaluation [20]. Hence, social marketing encourages the social behavioral change of the target audience. This social behavioral change refers to the behavior of a certain number of target audiences, not just individual behavior. Moreover, the final goal of social marketing behavioral change is not only to change the behavior of the target audiences, but also to keep the audience’s behavior after the change [21].

Social marketing activities include social concepts. Social concepts are not only different from tangible objects but also fundamentally different from intangible services, making the understanding of social marketing content more abstract [22]. It is often difficult for the marketing subject to accurately and specifically describes the characteristics of its marketing purpose, which brings many difficulties to the promotion of marketing activities, making it difficult to communicate effectively with the target audience [23]. Therefore, it is necessary to develop a strategy by investigating the key criteria for the success of social marketing activities or campaign.

### 2.3. Social Marketing Success Criteria

The concept of a “success factor” or “success criterion” was first developed by Ronald in 1961 [24]. The success criterion is the concept often used to explore the relationship between industrial characteristics and corporate strategy in the combination of the special abilities and the corresponding important requirements of the environment in order to obtain good performance [24]. The concept was widely applied in different fields. In the case of organizations, the key success criterion approach is to identify the key criteria that make the organization successful through analysis and then to determine and plan around these key criteria [25]. According to Kotler et al. [20], social marketing success criteria are those results-driven criteria that deserve more attention from the practitioner during the social campaign. The proposed fourteen key success criteria and the arguments of scholars are discussed in the following sections.

### 2.4. Success Leading Criteria

The fourteen success criteria are: Marketing Mix Elements (C1), Appropriate Media (C2), Designing an Effective Communication Message (C3), Feedback System (C4), Meeting the Needs of Beneficiaries (C5), Providing More Benefit than Cost (C6), Consumer Orientation (C7), Long-Term Planning (C8), Operation Process (C9), Information Resources (C10), Market Selection (C11), Moderate Research (C12), Monitoring and Adjustment (C13), Organizational Advantage (C14). The discussions of each criterion are as follows.

#### 2.4.1. Marketing Mix Elements

The components of social marketing mix strategy are product, price, place, promotion, public, policy, partnerships and purse strings. Lucz and Suggs [26] identified these and argue that the strategies used in the social marketing mix in health behavior change interventions must be implemented to ensure its success.

#### 2.4.2. Appropriate Media

Successful social marketing campaigns make good use of media channels and spokespersons to efficiently communicate to the target audience. A social marketer should ensure that the target audience receives and understands the message by means of appropriate media [27].

#### 2.4.3. Designing an Effective Communication Message

Effective communication message is motivated by the objective of the campaign. Within social marketing campaign strategy, the communication strategy expresses how to capture the target audiences’ attention and transmit a persuasive campaign message. The most critical factor in social marketing is to design an attractive message to encourage the target audience to adopt the health-promoting behavior [28].

#### 2.4.4. Feedback System

Feedback systems are used to control and regulate processes. Successful social marketing would establish a feedback mechanism in order to modify or adjust the plan in order to keep the campaign in a state of progress [29].

#### 2.4.5. Meeting the Needs of Beneficiaries

Every social marketing campaign needs to consider the condition of the target audience. A comprehensive approach must be taken to meet the needs of beneficiaries by understanding the cultural differences, values and beliefs of the target audience for the social marketing campaign to be accessible [30].

#### 2.4.6. Providing More Benefit than Cost

One of the reasons consumers buy a product considers the cost-benefit of the product. For the same reason, the product introduced by social marketing is a process of behavioral change. Therefore, considering the cost-benefit ratio of the behavioral change would reduce the barriers to change and, consequently, provide more benefits of changing; thus, the behavior would increase the willingness to change. Any social marketing campaign that intends to motivate the behavior has to consider the relative cost and the benefit of the competing products [7].

#### 2.4.7. Consumer Orientation

Customer orientation is a social marketing factor that requires the social marketing practitioner to focus on the behavioral change [31]. It is an organizational-wide philosophy that the target audience’s wants and needs are the first priority of the social marketing campaign.

#### 2.4.8. Long-Term Planning

Long-term planning is about setting an activity that begins now and continues well into the future, by which a particular strategic plan will be achieved. It involves establishing periodical goals that the social campaign is expected to achieve within certain period of time [32].

#### 2.4.9. Operation Process

In many situations, people are willing to change certain health behavior, provided the process is simple and easy to accomplish. The social marketing campaign should make the process of behavioral change simpler, feasible and convenient in order to encourage the actual performance of the target audience [33].

#### 2.4.10. Information Resources

The failure of some activities is often due to a lack of proper publicity, resulting in the target audience knowing less about the campaign message. It is an important decision to allocate information resources with reference to the integrated health communication strategy in social marketing campaigns [34].

#### 2.4.11. Market Selection

The reason why some social marketing campaigns fail is due to the target audience not recognizing the problems or issues that need to be solved. Thus, the chances of success may be increased if the social marketing campaign targets the specific audience who is ready for the behavioral change [35].

#### 2.4.12. Moderate Research

The use of moderate research in designing social marketing campaigns may increase the effectiveness and overall positive influence. Moderate research in advance will help to clarify the answers to many questions [36].

#### 2.4.13. Monitoring and Adjustment

It is important to develop the monitoring and adjustment plan before starting any social activities so that there is a preparation for what questions about the campaign need to be answered. Successful social marketing establishes a monitoring and adjustment mechanism to modify or adjust the relevant objectives and actions to keep the plan in a progressive state [37].

#### 2.4.14. Organizational Advantage

Organizational advantage accrues from the particular capabilities organizations have for creating and sharing knowledge [38]. Charisma of the leader and passion of the volunteers are the positive strengths for improving the image of non-profit organization and gaining organizational advantage.

### 2.5. Social Marketing in Health Promotion

Health promotion campaigns are used to raise awareness about health issues with the aim of improving health outcomes and community wellbeing. However, knowing how to plan and where to start these campaigns may not be easy. Health promotion requires marketing an effective message to foster the targeted audience to adopt new behaviors [39]. Health promotion applies various techniques that make a contribution to wellbeing, with social marketing playing an increasingly important role [40]. Thackeray and Brown [41] also claimed that social marketing makes unique contributions to health promotion practices. Thus, social marketing has the ability to increase the effectiveness of health improvement work, with the intention that it should build on core health promotion principles [42]. Da Silva and Mazzon [43] also argue that it is important to develop social marketing as to discuss the factors that improve the success of health promotion. Perez-Mujica et al. [44] suggested implementing an agent-based model into the design of social marketing campaigns for health promotion.

There are various principles associated with the integration of the social marketing success criteria in health promotion [45]. It will be valuable for practitioners to identify those success criteria to improve social marketing performances. Nevertheless, health promotion practitioners also strive to figure out the approach for successful implementation of the health promotion concept in their respective areas. Therefore, this research aims to evaluate the criteria associated with the effective implementation of social marketing from the health promotion perspective. Furthermore, this research provided the causal relation and ranks the fourteen criteria from the prominence in order to guide the health promotion practitioner through the selection of the criteria when they have limited access to resources.

### 2.6. Respondents

Tzu Chi Foundation is a Taiwan Buddhism charity non-profit organization founded by Dharma Master Cheng Yen in 1966. Aside from promoting the concepts and activities of environmental protection (reuse, reduce and recycle), Tzu Chi foundation also established several schools and universities to expand and deepen the effectiveness of the education. Although, the foundation built several hospitals in Taiwan, the founder also wanted the volunteers to encourage the concept of health-promoting lifestyle to the public. The volunteers are an extremely important resource in the activities of health promotion, as they are physically involved in promoting healthy lifestyle by adopting a vegetarian diet. Moreover, the volunteers also organize events and activities such as group exercise and chronic disease health management campaigns. These participants should be qualified with the following features: (a) having managerial position or major role in the health promotion and (b) having at least 15 years of experience in promoting health concepts.

## 3. Materials and Methods

### 3.1. DEMATEL Method

The DEMATEL method was developed by the Banelle Institute of Geneva for the Sciences and Human Affairs Program between 1972 and 1976 [46]. The method is used to solve complex entanglements, and it can be used to improve the understanding of specific cluster problems and to provide feasible solutions by providing a hierarchical structure [47]. The most significant aspect of DEMATEL is the function of constructing the relation and structure of the criteria in multi-factors decision making area [48,49,50]. In addition, the DEMATEL method was successfully applied in many situations, such as marketing strategy, social marketing, environmental issues, information systems, service quality and in the business ecosystem [51,52,53,54]. However, the problem of the organization is mostly under uncertain circumstances. As the human-centric activity is processed, the DEMATEL method would not be applicable or appropriate for solving the multiple-factor variable in an uncertain situation. Hence, it is necessary to build an extend DEMATEL method by applying a fuzzy theory.

### 3.2. Fuzzy Theory

The fuzzy theory refers to the use of fuzzy mathematical methods to deal with some complex decision-making problems [55]. Such problems generally have the characteristics of large systems. The relationship between the systems is very complicated, and these variables cannot be accurately assigned. These variables are fuzzy factors and involve certain subjective elements, making the relationship between subsystems and variables unclear [56]. Therefore, it must be dealt with by methods such as ranking and fuzzy evaluation. In real life, many concepts are vague. Fuzzy decision-making theory is a very practical tool in solving these vague and complex problems.

### 3.3. F-DEMATEL

The use of a fuzzy theory includes adapting the DEMATEL procedure for vague and imprecise data. The F-DEMATEL method had to be developed according to the expert’s opinion with linguistic variables. This fuzzy decision method has been verified as effective in congregating the viewpoints of professionals and thus providing information of greater reliability in various areas. Applications of F-DEMATEL are popular as the method is applied in many areas such as the selection of research and development projects [57], the identification of factors in emergency management [48], the determination of sustainable project management in construction and the selection criteria of suppliers [58,59].

In this research, F-DEMATEL was applied in order to compare the relationship between the attributes of criteria directly. The matrix operation is used to find out the direct and indirect causal relationship and influence intensity of all criteria attributes, especially by using the matrix and matrix diagram of visual structure to express the causal relationship and influence degree between the criteria attributes in complex systems, assisting in the decision-making [60]. Thus, F-DEMATEL converted a complex system into a structural causal relationship. The relationship between criteria attributes in complex systems is reduced to cause and effect groups, through the degree of interrelation between quantitative criteria attributes, to help the identification of the core problems of complex systems and the direction of improvement, which also improves the construct’s validity [9]. This research applied F-DEMATEL to evaluate the complex interrelation success criteria of social marketing in order to effectively implement health promotion. Moreover, in many situations, the DEMATEL directly adopts crisp values to assess the degree of influence among criteria, which ignores the fact the experts generally make fuzzy linguistic measurement according to the experiences instead of crisp value [59]. Hence, F-DEMATEL method is the most suitable method to be applied in order to reduce uncertainty in the subjective evaluation by experts and, at the same time, to increase the representative reliability in this research. Lastly, F-DEMATEL method also improved the surface validity, which means that the academic circle determined criteria that really measures the structure. As mentioned earlier, the F-DEMATEL method is commonly applied in fields of manufacturing, management, project development and many others science domains for decision making purposes.

### 3.4. Description of F-DEMATEL

A brief description of the computation procedures of F-DEMATEL is presented in the following sections as individual steps [54,55].

#### 3.4.1. Step 1: Select and Collect the Viewpoint of the Experts on the Research Issue

The research aimed to identify the criteria affecting the social marketing campaign for promoting a healthy base from the survey answers of the participants. Twelve health promotion participants answered the survey questionnaire. In order to increase the stability and reliability, this research used the test-retest method to examine the stability of an indicator. The procedure is to test and retest the same indicator to the same group of experts, if the same or close result is obtained each time, then the indicator has a stable degree of confidence [61].

#### 3.4.2. Step 2: Design the Fuzzy Linguistic Scale 

**Figure 1:**
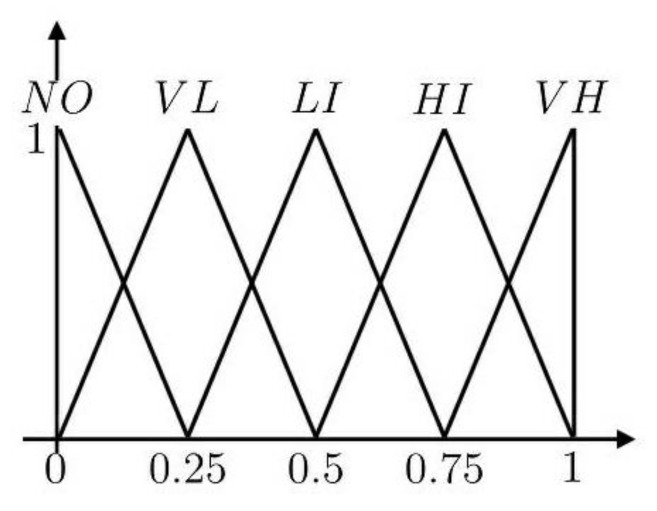
Triangular fuzzy numbers for linguistic variables. VH, Very high influence; HI, High influence; LI, Low influence; VL, Very low influence; NO, No influence.

A linguistic variable is a linguistic expression labeling granular information. The values of the variable are either words or sentences in natural or artificial language. Such linguistic valuations are vague, which makes further analysis difficult to compute. This study turned the ambiguous value into triangular fuzzy numbers based on Table 1. Figure 1 details the triangular fuzzy numbers for linguistic variables in general.

#### 3.4.3. Step 3: Compute the Initial Direct-Relation Fuzzy Matrix

Let i=1,2,3,…,n be *n* evaluation criteria. The p experts are requested to compare criteria in pairs to develop $Z^{\left(1\right)},\;Z^{\left(2\right)},\ldots ,Z^{\left(p\right)}.$ The fuzzy matrix $Z^{\left(k\right)}$ is the initial direct-relation fuzzy matrix of expert k, followed by Equation (1)

(1) $$Z^{\left(k\right)}=\left[\begin{array}{cccc} 0 & Z_{12}^{\left(k\right)} & \cdots & Z_{1n}^{\left(k\right)}\\ Z_{21}^{\left(k\right)} & 0 & \cdots & Z_{2n}^{\left(k\right)}\\ \vdots & \vdots & \ddots & \vdots \\ Z_{n1}^{\left(k\right)} & Z_{n2}^{\left(k\right)} & \cdots & 0 \end{array}\right]\;k=1,2,\ldots ,p$$

where $\;Z_{ij}^{\left(k\right)}=\left(l_{ij}^{\left(k\right)},\;m_{ij}^{\left(k\right)},\;u_{ij}^{\left(k\right)}\right)\;$.

#### 3.4.4. Step 4: Calculate the Normalize Direct-Relation Fuzzy Matrix

(2) $$r^{\left(k\right)}=\underset{1\;\leq \;i\;\leq n\;}{\max}\left(\sum_{j=1}^{n}u_{ij}^{\left(k\right)}\right)\;k=1,2,\ldots ,p$$

The linear scale transformation is applied in order to compare the criteria. Follow by acquiring the normalized direct-relation fuzzy matrix as $X^{\left(k\right)}$

(3) $$X^{\left(k\right)}\;=\;\left[\begin{array}{cccc} X_{11}^{\left(k\right)} & X_{12}^{\left(k\right)} & \cdots & X_{1n}^{\left(k\right)}\\ X_{21}^{\left(k\right)} & X_{22}^{\left(k\right)} & \cdots & X_{2n}^{\left(k\right)}\\ \vdots & \vdots & \ddots & \vdots \\ X_{n1}^{\left(k\right)} & X_{n2}^{\left(k\right)} & \cdots & X_{nn}^{\left(k\right)} \end{array}\right]\;k=1,2,\ldots ,p$$

where $X_{ij}^{\left(k\right)}=\left(L_{ij}^{\left(k\right)},M_{ij}^{\left(k\right)},U_{ij}^{\left(k\right)}\;\right)=\left(\frac{Z_{ij}^{\left(k\right)}}{r^{\left(k\right)}}\right)=\left(\frac{l_{ij}^{\left(k\right)}}{r^{\left(k\right)}},\;\frac{m_{ij}^{\left(k\right)}}{r^{\left(k\right)}},\frac{u_{ij}^{\left(k\right)}}{r^{\left(k\right)}}\right)$.

Equation (4) is used to find the average matrix of $X^{\left(k\right)}$ for k=1,2,…,p

(4) $$L=\;\left[\begin{array}{ccc} L_{11} & \cdots & L_{1n}\\ \vdots & \ddots & \vdots \\ L_{n1} & \cdots & L_{nn} \end{array}\right],M=\;\left[\begin{array}{ccc} M_{11} & \cdots & M_{1n}\\ \vdots & \ddots & \vdots \\ M_{n1} & \cdots & M_{nn} \end{array}\right],U=\;\left[\begin{array}{ccc} U_{11} & \cdots & U_{1n}\\ \vdots & \ddots & \vdots \\ U_{n1} & \cdots & U_{nn} \end{array}\right]$$

where $L_{ij}=\frac{1}{p}\overset{p}{\underset{k=1}{\sum }}L_{ij}^{\left(k\right)},M_{ij}=\frac{1}{p}\overset{p}{\underset{k=1}{\sum }}M_{ij}^{\left(k\right)},U_{ij}=\frac{1}{p}\overset{p}{\underset{k=1}{\sum }}U_{ij}^{\left(k\right)}$

#### 3.4.5. Step 5: Generate and Analyze Structure Model

The total-relation fuzzy matrix T can be attained after normalizing direct-relation fuzzy matrix. It was proved that $\underset{n\to \infty }{\lim}U^{n}=0$ and then $\underset{n\to \infty }{\lim}L^{n}=\underset{n\to \infty }{\lim}M^{n}=0$. The total-relation fuzzy matrix is calculated using Equation (5) to Equation (8).

(5) $$TL=\left[TL_{ij}\right]=\underset{c\to \infty }{\lim}\left(L+L^{2}+\cdots +L^{c}\right)=L{\left(I-L\right)}^{-1}$$

(6) $$TM=\left[TM_{ij}\right]=\underset{c\to \infty }{\lim}\left(M+M^{2}+\cdots +M^{c}\right)=M{\left(I-M\right)}^{-1}$$

(7) $$TU=\left[TU_{ij}\right]=\underset{c\to \infty }{\lim}\left(U+U^{2}+\cdots +U^{c}\right)=U{\left(I-U\right)}^{-1}$$

(8) $$T=\;\left[\begin{array}{cccc} \left(TL_{11},TM_{11},TU_{11}\right) & \left(TL_{12},TM_{12},TU_{12}\right) & \cdots & \left(TL_{1n},TM_{1n},TU_{1n}\right)\\ \left(TL_{21},TM_{21},TU_{21}\right) & \left(TL_{22},TM_{22},TU_{22}\right) & \cdots & \left(TL_{2n},TM_{2n},TU_{2n}\right)\\ \vdots & \vdots & \ddots & \vdots \\ \left(TL_{n1},TM_{n1},TU_{n1}\right) & \left(TL_{n2},TM_{n2},TU_{n2}\right) & \cdots & \left(TL_{nn},TM_{nn},TU_{nn}\right) \end{array}\right]$$

#### 3.4.6. Step 6: Obtained the Defuzzification Value of Total-Relation Fuzzy Matrix

The best non-fuzzy performance (BNP) method is applied to get the defuzzification values of $D_{i}$ and $R_{i}$ [47]. BNP value for triangular fuzzy number a=(l,m,u) can be calculated using Equation (9). The defuzzification values of total-relation fuzzy matrix can be obtained by Equation (10).

(9) $$\mathrm{BNP}=l+\frac{\left(u-l\right)+\left(m-l\right)}{3}\;\;\;\;$$

(10) $$\;T\;\prime =\;\left[\begin{array}{cccc} T_{11}^{\prime } & T_{12}^{\prime } & \cdots & T_{1n}^{\prime }\\ T_{21}^{\prime } & T_{22}^{\prime } & \cdots & T_{2n}^{\prime }\\ \vdots & \vdots & \ddots & \vdots \\ T_{n1}^{\prime } & T_{n2}^{\prime } & \cdots & T_{nn}^{\prime } \end{array}\right]\;\mathrm{where}\;T_{ij}^{\prime }=TL_{ij}+\frac{\left(TU_{ij}-TL_{ij}\right)+\left(TM_{ij}-TL_{ij}\right)}{3}\;\;$$

#### 3.4.7. Step 7: Establish and Analyze the F-DEMATEL Diagram

In Equations (11) and (12), the summation of columns and rows are plotted as vectors $D_{i}$ and $R_{i}$ accordingly. The horizontal axis vector $\left(D_{i}+R_{i}\right)$ known as “Prominence” is attained by add up $D_{i}$ to $R_{i}$, which determines the weight of each criterion. Likewise, the vertical axis $\left(D_{i}-R_{i}\right)$ termed by subtracting “Relation” is calculated $D_{i}$ from $R_{i}$. Then this classifies the criteria into cause and effect sets. When $\left(D_{i}-R_{i}\right)$ is positive, it means that the criterion is the cause factor. The criterion is the effect one when $\left(D_{i}-R_{i}\right)$ is negative.

As a result, the causal model is drawn by plotting the dataset of (D+R, D−R).

(11) $$D_{i}\;=\sum_{x=1}^{n}T_{ix}^{\prime }$$

(12) $$R_{i}=\sum_{y=1}^{n}T_{yj}^{\prime }$$

## 4. Results 

The social marketing criteria influencing health promotion were analyzed and constructed using F-DEMATEL method. The initial direct-relation fuzzy matrix indicated in Table 2 and Table 3 were attained through Equation (1). 

**Figure 2:**
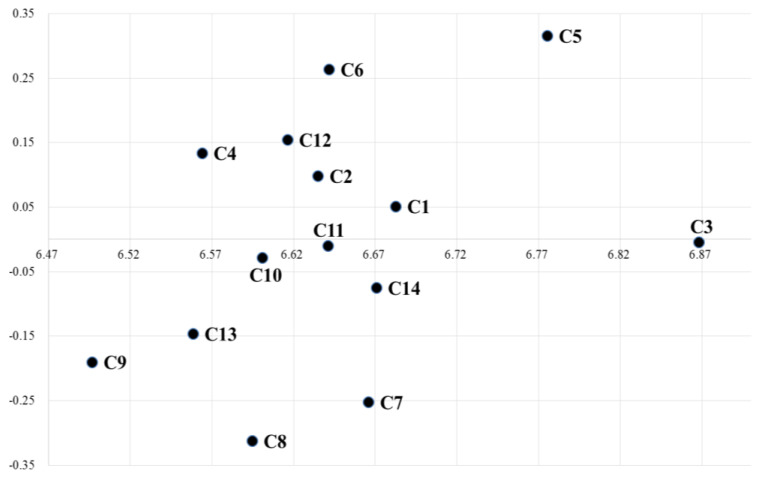
The cause and effect relationship diagram. C1, Marketing mix elements; C2, Appropriate media; C3, Designing effective communication message; C4, Feedback system; C5, Meeting the needs of beneficiaries; C6, Providing more benefit than cost; C7, Consumer orientation; C8, Long-term planning; C9, Operation Process; C10, Information resources; C11, Market Selection; C12, Moderate research; C13, Monitoring and adjustment; C14, Organizational advantage.

After indicating the initial direct-relation fuzzy matrix, the procedure continued by normalizing the direct-relation fuzzy matrix. The normalized direct-relation fuzzy matrix as shown in Supplementary Materials Tables S1–S3 can be obtained through Equations (2) and (3). As soon as the normalized fuzzy direct-relation matrix X is obtained, the total-relation fuzzy matrix *T* can be acquired by using Equations (4) to (8) as shown in Supplementary Materials Tables S4–S6. The defuzzification values of total relation fuzzy matrix are shown in Table 4. Then, the indexes of each criterion of defuzzification values of total relation fuzzy matrix can be acquired from Equations (9) and (10). The prominence and relation for cause and effect is obtained using Equations (11) and (12) as indicated in Table 5. According to the results, the diagram of the cause–effect relationship (Figure 2) is mapped based on the data sets of $\left(D_{i}+R_{i},\;D_{i}-R_{i}\right)$.

According to the outcomes, the criteria are organized and set according to the degree of the importance based on the $\left(D_{i}+R_{i}\right)$ value. Designing an effective message (C3) has the highest value among the criteria. The rest of the criteria are arranged as follow C5 > C1 > C14 > C7 > C6 > C11 > C2 > C12 > C10 > C8 > C4 > C13 > C9. The criteria are divided into two groups as shown in Figure 2, in order to identify whether the value of $\left(D_{i}-R_{i}\right)$ is positive or negative. The positive values of the cause group are C1, C2, C4, C5, C6 and C14. The rest of the negative values including C3, C7, C8, C9, C10, C11, C12 and C13 are the effect group. 

The criteria of the cause group are crucial because of their explicit influence on the effect group. Therefore, it would be substantial to discuss these criteria. Among all the cause group criteria, “Designing effective communication message” (C3) has the highest $\left(D_{i}+R_{i}\right)$ value with 6.868. This implies that it is the most important criterion in social marketing. However, its $\left(D_{i}-R_{i}\right)$ value (equals to −0.005) is a negative value, which can be justified by the fact that “Designing effective communication message” is important among all but slightly influenced by other criteria. 

“Meeting the needs of beneficiaries” (C5) has the highest $\left(D_{i}-R_{i}\right)$ value with 0.316 followed by “Providing more benefit than cost” (C6), providing more benefit than cost with 0.264, which implies that these two criteria had more impact on the whole social marketing criteria. Moreover, the $\left(D_{i}+R_{i}\right)$ values of (C5) and (C6) equal to 6.775 and 6.642 are relatively high, which can be described as these two criteria having had high influence over the other criteria and being more important among all. Thus, “Meeting the needs of beneficiaries” and “Providing more benefit than cost” play an essential role in the social marketing strategy of health promotion. “Marketing mix elements” (C1) is considered important with the $\left(D_{i}+R_{i}\right)$ value 6.682 and the positive $\left(D_{i}-R_{i}\right)$ value 0.051, indicating that the marketing mixed strategy improves the success of social marketing campaign of health promotion.

“Moderate research” (C12) with $\left(D_{i}-R_{i}\right)$ value of 0.155 holds the third position, indicating its impact on the other criteria. This means that the success of social marketing also relies on moderate research. This specified that the key attribute of the social marketing strategy is the use of formative research [36]. The next sequence according to the influence on the other criteria can be enlisted as “feedback system” (C4) with the value of 0.134, also considered as the cause groups that help in getting the message returned by the beneficiaries. The “feedback system” secures the commitments of the target audience to implement the suggested health-promoting behavior. “Appropriate media” (C2) with $\left(D_{i}-R_{i}\right)$ value of 0.098 indicated that the selection of media helped to spread the effective message to the beneficiaries. The result implied that using precise media will maximize the impact of a social marketing campaign. 

Criteria in the effect group were influenced by the cause criteria. Nevertheless, some of these criteria require deliberation to explore their contribution in an overall manner. Among these criteria, “customer orientation” (C7) attains $\mathrm{the}\;\left(D_{i}-R_{i}\right)$ value of −0.252, which indicates that this criterion receives the impact from cause group. Moreover, it is the fifth criteria with the $\left(D_{i}+R_{i}\right)$ value of 6.666, signifying the importance of this criterion. “Marketing selection” (C11) also has the $\left(D_{i}-R_{i}\right)$ value of −0.010, but the $\left(D_{i}+R_{i}\right)$ value of 6.641 is moderately high, which implied that beginning with the participants who are most ready for the behavior change increased the success of the social marketing campaign. “Organizational advantage” (C14) should also be considered as important due to the $\left(D_{i}+R_{i}\right)$ value of 6.671, which is comparatively high. Although the $\left(D_{i}-R_{i}\right)$ value of −0.252 belongs to the effect group, it still signified that a well-known or famous organization will have more advantages promoting health concepts.

## 5. Discussion

The framework applied in this study would provide practical insights into the analysis of criteria to implement social marketing in promoting a healthy lifestyle. Based on the findings, “designing effective communication message” is the most important criteria. Tzu Chi foundation is one of the non-profit organizations that designs communication messages aiding in spreading its compassionate cause. Master Cheng Yen’s “Jing Si Aphorisms” encourage compassionate thoughts, leaving imprints in people’s hearts. There are many “Jing Si Aphorisms” messages promoting mental health and a healthy lifestyle. For example: “Kindness is a wisdom, a vision, a self-confidence, a spiritual strength, a culture, a happiness”; “Whole grain is a natural food of human beings, it leads to peaceful and harmonious life”; “Vegetarianism means disciplining ourselves, cherishing our own lives and respecting all living beings”; “Vegetarian diet fosters one’s endurance, compassion and wisdom” [62]. Master Cheng Yen stressed that spreading good words to “the entire street” is excellent behavior [63]. The result is in line with the indication of Key and Czaplewski [28] that the social marketer practitioner should design an attractive message to capture the attention of the target audience for behavioral change. This indicates that effective message approaches have a significant influence on the intentions of a health promotion strategy [64].

The other two criteria, “meeting the needs of beneficiaries” and “providing more benefit than cost”, are the second and seventh important criteria and the major influence criteria among all. Social marketing uses systematic marketing techniques to help and motivate the target audience to change their behavior voluntarily through a customer-oriented approach. This strategy is to generate self-interest in changing behavior among the target audience and, on that basis, to help them voluntarily change behavior and sustain it. Allowing the audience to change behavior voluntarily rather than forcibly is the main purpose of social marketing. This outcome is consistent with the research of Wymer [30] and Pettigrew [7] that the social marketing campaign in health promotion should provide more benefits and meet the needs of target audience in order to be successful. 

“Appropriate Media” belongs to both important and cause group. Tzu Chi foundation had its own television, broadcasting, magazine and internet social media to spread the message of the campaign, as well as the concept and practice of environmental protection and promotion of a healthy diet. Hence, the result concludes that communication is a central aspect of health promotion, and the opportunity for mass communication makes the media a popular option amongst practitioners [39]. Vadel et al. [65] suggested that for nonprofit organizations, implementing a social media strategy can help achieve health promotion goals. In connection with “designing effective communication message” and “meeting the needs of beneficiaries”, scholars concluded that social media holds promise as an effective health promotion mechanism; however, information must be reliable and composed of attractive messages customized to meet the needs of the target audience [66,67].

“Marketing mix elements” ranks as the third most important criteria and is also the influencial criteria. Moreover, “customer orientation”, “organizational advantage” and “market selection” are the top five important criteria but belong to the effect group. Hence, in order to accomplish a successful social marketing campaign in health promotion, the practitioner must consider the needs of the recipients and select those ready to engage the audience [35]. Moreover, the practitioner has to design an attractive message to communicate the benefits of the behavioral change to the target audience by meeting the needs of beneficiaries. Besides, the message delivered by the known organization, such as the Tzu Chi foundation, increased the success of social marketing campaign in promoting a healthy lifestyle and a vegetarian diet. Through the development of customer orientation [31], the practitioner should establish the location of the target audience, segment the market objective, position the target product or services, develop the marketing mix elements [26] and monitor the feedback system [29] to increase the success of the social marketing campaign in promoting a healthy lifestyle to the public. Purcarea [68] also recommended that the marketing mix strategy is necessary for health promotion to be effective; the target audience will have to be segmented correctly, and desirable messages will have to resonate with the target audience.

In contrast with the previous studies, the results of this research denoted that through systematic analysis of the social marketing steps, correct selection of the target object and a comprehensive understanding of it can result in well set goals and objectives. These goals and objectives are further sustained through the development of integration strategies, developing a strategy that can induce behavioral change and at the same time effectively motivate the target of the promotion strategy by establishing the indicators of the results intended to be implemented, determining that all development efforts necessary to ensure success. The influencing criteria plays an important role in the improvement of the campaign. Furthermore, it is important to understand the needs of the target audience for health promotion in order to develop social marketing strategies [69].

According to the results, some criteria were not listed as important or cause criteria. Nevertheless, the practitioner may still implement all of the criteria to further ensure the success of the campaign when there are sufficient resources, as claimed by Kotler and Lee [10].

## 6. Conclusions

This research has investigated fourteen social marketing success criteria based on the review of literature and opinions of scholars which are related to health promotion in the context of the non-profit organization sector. The result adds relevant contributions to the academic and the actual performance in the field of health promotion. The outcome of this study intends to guide the health promotion practitioner to focus their future efforts using the refined key success criteria of social marketing efficiently and productively. The itemized success criteria will help improve the social marketing performance in promoting a healthy lifestyle. The results of this study can further help health promotion professionals in understanding the health-promoting lifestyle and also aid in designing appropriate health promoting campaigns, thus improving the well-being and overall quality of life of the public and of the society. In fact, it is not hard to launch a social marketing campaign, but it is the biggest challenge to be able to convince the target audience. However, if the practitioner applies the above-mentioned social marketing key success criteria and makes sure that the campaign actually exerts social influence, it will be able to persuade the target audience to take action towards a healthy lifestyle. In most cases, there are limited resources for non-profit organizations, especially funds for social campaigns. Therefore, this study provides important information for non-profit organizations in selecting significant criteria that aid in the efficacy of such campaigns.

### 6.1. Implications of the F-DEMATEL Method

The F-DEMATEL method was undertaken to obtain the key success criteria of social marketing, as well as to improve the effectivity and the influence of health promotion implementation. This is the first research that used the F-DEMATEL method in the field of social marketing in promoting a healthy lifestyle. The method is not only rationally significant in measuring the success criteria of social marketing but also supportive in responding to the how and why in terms of relationships. The method also further obtained the causal relationships among different criteria in the social marketing strategy. These criteria are categorized as cause and effect groups towards the performance result. Moreover, the effects of the strategic results may be accomplished by continuously improving criteria of cause group as these criteria influence the strategy directly. Thus, working on the criteria of the cause group is substantial for practitioners. These will influence the criteria of effect group in order to improve the performance of social marketing strategies in health promotion.

### 6.2. Limitations and Recommendation

The F-DEMATEL method is widely applied in numerous areas. This is the first study applying this methodology in the field of social marketing and health promotion. However, this structural model has its own limitations. The model is highly reliant on the assessment of the experts. The effect of uncertainty and human bias in evaluating the criteria have to be considered further in future study. Future studies may also advance the research to identify the interlinked relations among social marketing success criteria using related techniques such as a modified F-DEMATEL method or a modified DEMATEL method.

## Figures and Tables

**Table 1:** The correspondence of linguistic terms and values.

Linguistic Terms	Linguistic Values
Very high influence	(0.75, 1.00, 1.00)
High influence	(0.50, 0.75, 1.00)
Low influence	(0.25, 0.50, 0.75)
Very low influence	(0.00, 0.25, 0.50)
No influence	(0.00, 0.00, 0.25)

**Table 2:** The initial direct-relations fuzzy matrix of expert 1 to 7.

Criteria	C1	C2	C3	C4	C5	C6	C7
**C1**	(0, 0, 0)	(0.036, 0.056, 0.071)	(0.038, 0.056, 0.071)	(0.034, 0.053, 0.066)	(0.026, 0.045, 0.062)	(0.029, 0.046, 0.061)	(0.037, 0.057, 0.071)
**C2**	(0.034, 0.052, 0.068)	(0, 0, 0)	(0.026, 0.046, 0.065)	(0.029, 0.049, 0.068)	{0.027, 0.048, 0.064}	(0.038, 0.057, 0.071)	(0.029, 0.049, 0.068)
**C3**	(0.03, 0.05, 0.066)	(0.041, 0.061, 0.076)	(0, 0, 0)	(0.041, 0.061, 0.075)	(0.037, 0.057, 0.074)	(0.029, 0.049, 0.066)	(0.028, 0.049, 0.066)
**C4**	(0.031, 0.052, 0.069)	(0.029, 0.049, 0.069)	(0.029, 0.049, 0.069)	(0, 0, 0)	(0.031, 0.048, 0.066)	(0.027, 0.046, 0.064)	(0.038, 0.058, 0.075)
**C5**	(0.038, 0.058, 0.07)	(0.043, 0.063, 0.077)	(0.031, 0.051, 0.068)	(0.027, 0.047, 0.066)	(0, 0, 0)	(0.037, 0.057, 0.074)	(0.039, 0.059, 0.074)
**C6**	(0.039, 0.059, 0.073)	(0.021, 0.04, 0.059)	(0.035, 0.056, 0.069)	(0.034, 0.054, 0.069)	(0.031, 0.049, 0.066)	(0, 0, 0)	(0.037, 0.057, 0.073)
**C7**	(0.027, 0.044, 0.061)	(0.026, 0.042, 0.061)	(0.038, 0.058, 0.073)	(0.02, 0.04, 0.059)	(0.034, 0.053, 0.066)	(0.029, 0.05, 0.068)	(0, 0, 0)
**C8**	(0.023, 0.04, 0.057)	(0.022, 0.042, 0.062)	(0.032, 0.052, 0.069)	(0.026, 0.046, 0.063)	(0.026, 0.044, 0.061)	(0.028, 0.048, 0.067)	(0.038, 0.058, 0.073)
**C9**	(0.025, 0.044, 0.061)	(0.031, 0.051, 0.068)	(0.03, 0.051, 0.068)	(0.017, 0.037, 0.056)	(0.029, 0.048, 0.065)	(0.022, 0.042, 0.063)	(0.027, 0.048, 0.065)
**C10**	(0.023, 0.04, 0.059)	(0.027, 0.048, 0.066)	(0.039, 0.06, 0.075)	(0.036, 0.055, 0.068)	(0.02, 0.039, 0.059)	(0.029, 0.049, 0.066)	(0.039, 0.06, 0.076)
**C11**	(0.036, 0.054, 0.068)	(0.04, 0.06, 0.072)	(0.038, 0.058, 0.075)	(0.036, 0.057, 0.07)	(0.031, 0.049, 0.068)	(0.026, 0.044, 0.061)	(0.0270.048, 0.066)
**C12**	(0.039, 0.058, 0.073)	(0.024, 0.043, 0.059)	(0.039, 0.059, 0.075)	(0.034, 0.055, 0.07)	(0.028, 0.049, 0.067)	(0.029, 0.046, 0.061)	(0.038, 0.058, 0.073)
**C13**	(0.042, 0.063, 0.075)	(0.036, 0.053, 0.067)	(0.018, 0.039, 0.059)	(0.022, 0.039, 0.058)	(0.037, 0.057, 0.072)	(0.026, 0.046, 0.064)	(0.029, 0.047, 0.064)
**C14**	(0.04, 0.06, 0.073)	(0.022, 0.041, 0.059)	(0.037, 0.057, 0.072)	(0.025, 0.045, 0.066)	(0.029, 0.049, 0.068)	(0.023, 0.044, 0.062)	(0.034, 0.052, 0.069)

C1, Marketing mix elements; C2, Appropriate media; C3, Designing effective communication message; C4, Feedback system; C5, Meeting the needs of beneficiaries; C6, Providing more benefit than cost; C7, Consumer orientation; C8, Long-term planning; C9, Operation Process; C10, Information resources; C11, Market Selection; C12, Moderate research; C13, Monitoring and adjustment; C14, Organizational advantage.

**Table 3:** The initial direct-relations fuzzy matrix of expert 8 to 14.

Criteria	C8	C9	C10	C11	C12	C13	C14
**C1**	(0.044, 0.063, 0.075)	(0.041, 0.058, 0.07)	(0.045, 0.065, 0.075)	(0.034, 0.054, 0.068)	(0.031, 0.049, 0.065)	(0.029, 0.048, 0.066)	(0.022, 0.04, 0.059)
**C2**	(0.045, 0.065, 0.078)	(0.032, 0.051, 0.066)	(0.036, 0.056, 0.069)	(0.023, 0.044, 0.061)	(0.037, 0.058, 0.071)	(0.029, 0.049, 0.068)	0(.036, 0.056, 0.073)
**C3**	(0.038, 0.056, 0.072)	(0.031, 0.048, 0.063)	(0.03, 0.05, 0.067)	(0.028, 0.049, 0.066)	(0.034, 0.054, 0.071)	(0.039, 0.06, 0.075)	(0.03, 0.05, 0.067)
**C4**	(0.034, 0.054, 0.073)	(0.036, 0.056, 0.07)	(0.02, 0.04, 0.061)	(0.032, 0.052, 0.069)	(0.028, 0.049, 0.069)	(0.032, 0.052, 0.071)	(0.034, 0.051, 0.07)
**C5**	(0.032, 0.052, 0.069)	(0.042, 0.062, 0.075)	(0.036, 0.057, 0.072)	(0.035, 0.056, 0.071)	(0.034, 0.055, 0.07)	(0.033, 0.053, 0.07)	(0.039, 0.059, 0.074)
**C6**	(0.037, 0.057, 0.072)	(0.034, 0.053, 0.066)	(0.036, 0.056, 0.071)	(0.034, 0.054, 0.069)	(0.032, 0.051, 0.066)	(0.039, 0.059, 0.073)	(0.046, 0.067, 0.078)
**C7**	(0.033, 0.054, 0.071)	(0.034, 0.054, 0.071)	(0.027, 0.047, 0.064)	(0.031, 0.051, 0.07)	(0.022, 0.042, 0.062)	(0.027, 0.045, 0.061)	(0.03, 0.051, 0.066)
**C8**	(0, 0, 0)	(0.026, 0.043, 0.061)	(0.021, 0.038, 0.056)	(0.03, 0.048, 0.065)	(0.033, 0.053, 0.072)	(0.027, 0.046, 0.063)	(0.031, 0.051, 0.068)
**C9**	(0.028, 0.048, 0.065)	(0, 0, 0)	(0.033, 0.053, 0.068)	(0.034, 0.055, 0.067)	(0.031, 0.052, 0.067)	(0.028, 0.048, 0.067)	(0.024, 0.045, 0.063)
**C10**	(0.039, 0.058, 0.073)	(0.032, 0.052, 0.071)	(0, 0, 0)	(0.034, 0.053, 0.068)	(0.024, 0.041, 0.06)	(0.035, 0.053, 0.07)	(0.025, 0.044, 0.062)
C11	(0.023, 0.044, 0.062)	(0.027, 0.047, 0.068)	(0.026, 0.046, 0.065)	(0, 0, 0)	(0.025, 0.046, 0.066)	(0.033, 0.053, 0.072)	(0.031, 0.051, 0.07)
**C12**	(0.028, 0.049, 0.068)	(0.03, 0.05, 0.069)	(0.027, 0.047, 0.066)	(0.034, 0.053, 0.07)	(0, 0, 0)	(0.034, 0.054, 0.071)	(0.039, 0.059, 0.074)
**C13**	(0.025, 0.046, 0.066)	(0.027, 0.045, 0.064)	(0.028, 0.049, 0.067)	(0.023, 0.042, 0.063)	(0.024, 0.044, 0.064)	(0, 0, 0)	(0.031, 0.051, 0.069)
**C14**	(0.032,0.051,0.069)	(0.033,0.052,0.067)	(0.039,0.06,0.076)	(0.035,0.056,0.074)	(0.024,0.041,0.059)	(0.027,0.044,0.061)	(0,0,0)

C1, Marketing mix elements; C2, Appropriate media; C3, Designing effective communication message; C4, Feedback system; C5, Meeting the needs of beneficiaries; C6, Providing more benefit than cost; C7, Consumer orientation; C8, Long-term planning; C9, Operation Process; C10, Information resources; C11, Market Selection; C12, Moderate research; C13, Monitoring and adjustment; C14, Organizational advantage.

**Table 4:** The total-relation matrix and the values of each criterion for cause and effect groups.

$T^{\;\prime }$	C1	C2	C3	C4	C5	C6	C7	C8	C9	C10	C11	C12	C13	C14
**C1**	0.194	0.243	0.254	0.238	0.232	0.231	0.255	0.260	0.249	0.252	0.244	0.236	0.242	0.236
**C2**	0.243	0.192	0.245	0.236	0.235	0.241	0.250	0.262	0.244	0.246	0.236	0.243	0.243	0.251
**C3**	0.245	0.252	0.206	0.249	0.247	0.238	0.252	0.259	0.245	0.246	0.245	0.244	0.256	0.249
**C4**	0.242	0.239	0.248	0.189	0.236	0.231	0.256	0.253	0.247	0.233	0.244	0.237	0.246	0.247
**C5**	0.257	0.259	0.260	0.243	0.200	0.251	0.268	0.262	0.263	0.257	0.257	0.250	0.257	0.263
**C6**	0.253	0.235	0.257	0.244	0.241	0.192	0.260	0.260	0.249	0.251	0.250	0.242	0.255	0.263
**C7**	0.228	0.225	0.247	0.219	0.230	0.227	0.194	0.244	0.238	0.230	0.235	0.222	0.231	0.236
**C8**	0.221	0.221	0.238	0.221	0.221	0.222	0.244	0.190	0.225	0.220	0.229	0.229	0.228	0.234
**C9**	0.224	0.229	0.237	0.213	0.224	0.218	0.235	0.236	0.185	0.232	0.233	0.227	0.231	0.229
**C10**	0.229	0.233	0.252	0.236	0.224	0.230	0.254	0.252	0.241	0.191	0.240	0.226	0.243	0.236
**C11**	0.242	0.244	0.253	0.239	0.235	0.227	0.245	0.242	0.239	0.236	0.193	0.232	0.245	0.244
**C12**	0.249	0.234	0.258	0.241	0.237	0.232	0.258	0.250	0.245	0.241	0.247	0.192	0.249	0.254
**C13**	0.243	0.233	0.230	0.219	0.234	0.223	0.239	0.238	0.231	0.233	0.228	0.224	0.192	0.237
**C14**	0.245	0.228	0.251	0.229	0.233	0.226	0.249	0.247	0.241	0.247	0.244	0.227	0.236	0.195

C1, Marketing mix elements; C2, Appropriate media; C3, Designing effective communication message; C4, Feedback system; C5, Meeting the needs of beneficiaries; C6, Providing more benefit than cost; C7, Consumer orientation; C8, Long-term planning; C9, Operation Process; C10, Information resources; C11, Market Selection; C12, Moderate research; C13, Monitoring and adjustment; C14, Organizational advantage.

**Table 5:** The prominence and relation for cause and effect.

	*D_i_*	*R_i_*	*D_i_ + R_i_*	*D_i_ − R_i_*
**C1**	3.367	3.315	6.682	0.051
**C2**	3.367	3.268	6.635	0.098
**C3**	3.432	3.436	6.868	−0.005
**C4**	3.349	3.215	6.564	0.134
**C5**	3.546	3.230	6.775	0.316
**C6**	3.453	3.189	6.642	0.264
**C7**	3.207	3.459	6.666	−0.252
**C8**	3.141	3.453	6.594	−0.312
**C9**	3.153	3.343	6.496	−0.190
**C10**	3.286	3.314	6.601	−0.028
**C11**	3.316	3.326	6.641	−0.010
**C12**	3.385	3.231	6.616	0.155
**C13**	3.206	3.352	6.558	−0.146
**C14**	3.298	3.373	6.671	−0.075

C1, Marketing mix elements; C2, Appropriate media; C3, Designing effective communication message; C4, Feedback system; C5, Meeting the needs of beneficiaries; C6, Providing more benefit than cost; C7, Consumer orientation; C8, Long-term planning; C9, Operation Process; C10, Information resources; C11, Market Selection; C12, Moderate research; C13, Monitoring and adjustment; C14, Organizational advantage.

## Supplementary Materials

The following are available online at https://www.mdpi.com/1660-4601/17/17/6317/s1, Table S1: The normalized direct-relation fuzzy matrix (lower value); Table S2: The normalized direct-relation fuzzy matrix (Middle value); Table S3: The normalized direct-relation fuzzy matrix (Upper value); Table S4: The total-relation fuzzy matrix (Lower Value); Table S5: The total-relation fuzzy matrix (Middle Value); Table S6: The total-relation fuzzy matrix (Upper Value).
