# Supplementary material for: Evaluating the Social Marketing Success Criteria in Health Promotion: A F-DEMATEL Approach
Source: Int J Environ Res Public Health. 2020 Aug 31;17(17):6317. doi: 10.3390/ijerph17176317 (PMC7503311; doi:10.3390/ijerph17176317)
Supplement: Supplementary file 1 [file ijerph-17-06317-s001.pdf]

International Journal of
Environmental Research and Public Health

MDPI

Appendix A

See Tables A1-A3

Table A1. The normalized direct-relation fuzzy matrix (lower value)

X_l	C1	C2	C3	C4	C5	C6	C7	C8	C9	C10	C11	C12	C13	C14
C1	0	0.036	0.0376	0.0345	0.0264	0.0289	0.0373	0.0447	0.0413	0.0448	0.0342	0.0309	0.0288	0.0215
C2	0.0337	0	0.0257	0.029	0.0273	0.0382	0.0288	0.045	0.0322	0.0359	0.0232	0.0324	0.0285	0.0361
C3	0.0302	0.0408	0	0.0408	0.0372	0.0291	0.0286	0.0379	0.0308	0.0303	0.0285	0.0339	0.0394	0.0301
C4	0.0317	0.0288	0.0288	0	0.031	0.0271	0.0378	0.034	0.0362	0.02	0.0321	0.0283	0.032	0.0345
C5	0.0382	0.0429	0.0309	0.0268	0	0.0373	0.039	0.032	0.0424	0.0361	0.0358	0.0344	0.0333	0.0391
C6	0.0391	0.0215	0.0354	0.034	0.0307	0	0.0371	0.0367	0.0341	0.0358	0.0339	0.0321	0.0388	0.0463
C7	0.0271	0.0259	0.038	0.0202	0.0342	0.0294	0	0.0336	0.0338	0.0271	0.0309	0.0216	0.0268	0.0308
C8	0.0234	0.0217	0.0321	0.026	0.0257	0.028	0.038	0	0.0256	0.0206	0.0299	0.0331	0.0273	0.0309
C9	0.0253	0.0311	0.0304	0.017	0.0292	0.0223	0.0276	0.0281	0	0.0328	0.0347	0.0315	0.0279	0.0242
C10	0.0232	0.0272	0.0394	0.0366	0.0199	0.0289	0.0396	0.0395	0.0321	0	0.0342	0.0241	0.0346	0.0251
C11	0.0359	0.0402	0.038	0.0362	0.0308	0.0257	0.0274	0.0233	0.0272	0.0257	0	0.0253	0.0328	0.031
C12	0.0389	0.0238	0.0392	0.0346	0.0282	0.029	0.0381	0.0286	0.0302	0.0273	0.0343	0	0.0342	0.0392
C13	0.0426	0.0363	0.0185	0.0226	0.0368	0.0258	0.0291	0.0254	0.0266	0.0284	0.0235	0.024	0	0.0307
C14	0.0399	0.0218	0.0368	0.0252	0.0292	0.0235	0.0339	0.0324	0.0338	0.0397	0.0356	0.0238	0.0271	0

Table A2. The normalized direct-relation fuzzy matrix (Middle value)

X_m	C1	C2	C3	C4	C5	C6	C7	C8	C9	C10	C11	C12	C13	C14
C1	0	0.0564	0.0563	0.0532	0.0451	0.0461	0.0578	0.0635	0.0584	0.0653	0.0547	0.0497	0.0476	0.0402
C2	0.0524	0	0.0462	0.0495	0.0478	0.057	0.0493	0.0655	0.0509	0.0564	0.0437	0.0511	0.049	0.0566
C3	0.0507	0.0613	0	0.0612	0.0577	0.0496	0.0491	0.0568	0.0479	0.0508	0.049	0.0544	0.0599	0.0505
C4	0.0522	0.0493	0.0493	0	0.0481	0.046	0.0583	0.0545	0.0567	0.0405	0.0526	0.0488	0.0525	0.0516
C5	0.0587	0.0634	0.0514	0.0473	0	0.0578	0.0595	0.0524	0.0629	0.0566	0.0563	0.0549	0.0538	0.0596
C6	0.0596	0.0401	0.0558	0.0545	0.0492	0	0.0576	0.0572	0.0528	0.0562	0.0544	0.0508	0.0593	0.0668
C7	0.0441	0.0427	0.0585	0.0407	0.0531	0.0499	0	0.0541	0.0543	0.0476	0.0513	0.0421	0.0455	0.0512
C8	0.0402	0.0422	0.0526	0.0465	0.0446	0.0485	0.0585	0	0.0428	0.0377	0.0488	0.0536	0.046	0.0514
C9	0.0439	0.0516	0.0509	0.0374	0.048	0.0428	0.0481	0.0486	0	0.0533	0.0551	0.052	0.0483	0.0447
C10	0.04	0.0477	0.0599	0.0554	0.0387	0.0494	0.0601	0.0582	0.0525	0	0.0531	0.0412	0.0533	0.0438
C11	0.0546	0.0607	0.0585	0.0567	0.0494	0.0443	0.0479	0.0438	0.0477	0.0461	0	0.0458	0.0533	0.0515
C12	0.0576	0.0427	0.0597	0.055	0.0487	0.0458	0.0586	0.0491	0.0507	0.0478	0.053	0	0.0546	0.0597
C13	0.0631	0.0534	0.039	0.0396	0.0573	0.0463	0.0478	0.0459	0.0454	0.0489	0.0422	0.0444	0	0.0512
C14	0.0604	0.0406	0.0573	0.0457	0.0497	0.044	0.0525	0.051	0.0525	0.0602	0.0561	0.0406	0.0439	0

Table A3. The normalized direct-relation fuzzy matrix (Upper value)

X_u	C1	C2	C3	C4	C5	C6	C7	C8	C9	C10	C11	C12	C13	C14
C1	0	0.0717	0.0716	0.0666	0.0623	0.0613	0.0714	0.0751	0.0701	0.0752	0.0682	0.065	0.0661	0.0591
C2	0.0679	0	0.065	0.068	0.0647	0.0719	0.0679	0.0786	0.0661	0.0698	0.0608	0.0663	0.0678	0.0734

**C3**	0.0661	0.0767	0	0.0749	0.0748	0.0666	0.0662	0.072	0.0617	0.0679	0.0661	0.0714	0.075	0.0677
**C4**	0.0692	0.0698	0.0698	0	0.0667	0.0646	0.075	0.0731	0.0701	0.061	0.0696	0.0693	0.0712	0.0686
**C5**	0.0704	0.077	0.0684	0.0662	0	0.0747	0.0747	0.0694	0.0749	0.0719	0.0715	0.07	0.0709	0.0748
**C6**	0.0731	0.059	0.0697	0.0697	0.0662	0	0.073	0.0728	0.0666	0.0716	0.0697	0.0644	0.0729	0.0785
**C7**	0.061	0.0613	0.0737	0.0594	0.0667	0.0685	0	0.0712	0.0714	0.0646	0.0699	0.0626	0.0608	0.0665
**C8**	0.0573	0.0627	0.0697	0.0633	0.0615	0.0671	0.0737	0	0.0616	0.0565	0.0657	0.0722	0.063	0.0683
**C9**	0.061	0.0684	0.0681	0.0562	0.0651	0.0633	0.0651	0.0654	0	0.0686	0.067	0.0672	0.0669	0.0633
**C10**	0.0589	0.0663	0.0752	0.0686	0.0591	0.0665	0.0767	0.0732	0.0714	0	0.0684	0.0598	0.07	0.0608
**C11**	0.068	0.0722	0.0755	0.0702	0.068	0.0613	0.0665	0.0627	0.0682	0.0649	0	0.0663	0.0719	0.0701
**C12**	0.0728	0.0596	0.0749	0.0702	0.0676	0.061	0.0735	0.0678	0.0695	0.0664	0.07	0	0.0715	0.0748
**C13**	0.0749	0.067	0.0595	0.0582	0.0722	0.0649	0.0646	0.0664	0.0643	0.0677	0.0627	0.0649	0	0.0698
**C14**	0.0736	0.0595	0.0722	0.0662	0.0684	0.0627	0.0693	0.0696	0.0679	0.0769	0.0748	0.0592	0.0608	0

See Tables B1-B3

Table B1. The total-relation fuzzy matrix (Lower Value)

l''_ij	C1	C2	C3	C4	C5	C6	C7	C8	C9	C10	C11	C12	C13	C14
**C1**	0	0.036	0.0376	0.0345	0.0264	0.0289	0.0373	0.0447	0.0413	0.0448	0.0342	0.0309	0.0288	0.0215
**C2**	0.0337	0	0.0257	0.029	0.0273	0.0382	0.0288	0.045	0.0322	0.0359	0.0232	0.0324	0.0285	0.0361
**C3**	0.0302	0.0408	0	0.0408	0.0372	0.0291	0.0286	0.0379	0.0308	0.0303	0.0285	0.0339	0.0394	0.0301
**C4**	0.0317	0.0288	0.0288	0	0.031	0.0271	0.0378	0.034	0.0362	0.02	0.0321	0.0283	0.032	0.0345
**C5**	0.0382	0.0429	0.0309	0.0268	0	0.0373	0.039	0.032	0.0424	0.0361	0.0358	0.0344	0.0333	0.0391
**C6**	0.0391	0.0215	0.0354	0.034	0.0307	0	0.0371	0.0367	0.0341	0.0358	0.0339	0.0321	0.0388	0.0463
**C7**	0.0271	0.0259	0.038	0.0202	0.0342	0.0294	0	0.0336	0.0338	0.0271	0.0309	0.0216	0.0268	0.0308
**C8**	0.0234	0.0217	0.0321	0.026	0.0257	0.028	0.038	0	0.0256	0.0206	0.0299	0.0331	0.0273	0.0309
**C9**	0.0253	0.0311	0.0304	0.017	0.0292	0.0223	0.0276	0.0281	0	0.0328	0.0347	0.0315	0.0279	0.0242
**C10**	0.0232	0.0272	0.0394	0.0366	0.0199	0.0289	0.0396	0.0395	0.0321	0	0.0342	0.0241	0.0346	0.0251
**C11**	0.0359	0.0402	0.038	0.0362	0.0308	0.0257	0.0274	0.0233	0.0272	0.0257	0	0.0253	0.0328	0.031
**C12**	0.0389	0.0238	0.0392	0.0346	0.0282	0.029	0.0381	0.0286	0.0302	0.0273	0.0343	0	0.0342	0.0392
**C13**	0.0426	0.0363	0.0185	0.0226	0.0368	0.0258	0.0291	0.0254	0.0266	0.0284	0.0235	0.024	0	0.0307
**C14**	0.0399	0.0218	0.0368	0.0252	0.0292	0.0235	0.0339	0.0324	0.0338	0.0397	0.0356	0.0238	0.0271	0

Table B2. The total-relation fuzzy matrix (Middle Value)

m''_ij	C1	C2	C3	C4	C5	C6	C7	C8	C9	C10	C11	C12	C13	C14
**C1**	0.1017	0.1523	0.1584	0.1479	0.1397	0.1393	0.161	0.1656	0.1571	0.1621	0.1531	0.1429	0.1462	0.141
**C2**	0.1494	0.096	0.1466	0.142	0.1397	0.1468	0.1508	0.1648	0.1478	0.1518	0.1408	0.1418	0.145	0.1534
**C3**	0.1515	0.1576	0.1057	0.1559	0.1522	0.1434	0.1541	0.1605	0.1486	0.15	0.1488	0.148	0.1583	0.1515
**C4**	0.1471	0.1413	0.1471	0.0927	0.1382	0.1348	0.1565	0.1525	0.151	0.1353	0.1467	0.1377	0.1459	0.1467
**C5**	0.1636	0.1641	0.16	0.148	0.1022	0.1553	0.1685	0.1617	0.1672	0.1605	0.1605	0.1529	0.1575	0.1645
**C6**	0.1614	0.1402	0.161	0.1517	0.1464	0.0977	0.1638	0.1628	0.1551	0.1571	0.1559	0.1464	0.1596	0.168
**C7**	0.1363	0.1321	0.152	0.1288	0.1395	0.1352	0.0978	0.1486	0.1454	0.1384	0.1422	0.1285	0.1364	0.1431
**C8**	0.1298	0.1284	0.1435	0.1311	0.1289	0.131	0.1499	0.094	0.1318	0.1262	0.1368	0.1359	0.1338	0.1403

**C9**	0.1347	0.1387	0.1437	0.1245	0.1335	0.1274	0.1422	0.1421	0.0924	0.1421	0.1441	0.136	0.1375	0.1356
**C10**	0.1348	0.1387	0.1556	0.1442	0.1287	0.1368	0.1568	0.1547	0.146	0.0948	0.1459	0.1298	0.1458	0.1385
**C11**	0.1496	0.152	0.1555	0.1468	0.1396	0.1335	0.1472	0.1432	0.1431	0.1407	0.0968	0.1351	0.147	0.1468
**C12**	0.1554	0.1385	0.16	0.1482	0.1419	0.1376	0.1601	0.151	0.1489	0.1452	0.1504	0.0941	0.1512	0.1572
**C13**	0.1524	0.1405	0.1329	0.1264	0.1418	0.1308	0.1422	0.1401	0.1362	0.1386	0.1326	0.1292	0.0913	0.1416
**C14**	0.1535	0.133	0.1538	0.1361	0.1388	0.1323	0.1505	0.1487	0.1466	0.1525	0.1492	0.1295	0.1376	0.0966

Table B3. The total-relation fuzzy matrix (Upper Value)

u''_ij	**C1**	**C2**	**C3**	**C4**	**C5**	**C6**	**C7**	**C8**	**C9**	**C10**	**C11**	**C12**	**C13**	**C14**
**C1**	0.4742	0.5401	0.5614	0.5286	0.5279	0.5225	0.5632	0.5665	0.5447	0.5487	0.5435	0.5278	0.5439	0.5409
**C2**	0.5404	0.4754	0.5581	0.5322	0.5324	0.5342	0.5628	0.5721	0.5436	0.5465	0.5397	0.5312	0.5476	0.5561
**C3**	0.549	0.5568	0.5074	0.5481	0.5512	0.5393	0.5717	0.5767	0.5499	0.5549	0.5543	0.5456	0.5643	0.5614
**C4**	0.5465	0.5457	0.5674	0.4733	0.5391	0.5325	0.574	0.5723	0.5521	0.5437	0.5523	0.5388	0.5557	0.5569
**C5**	0.5661	0.5705	0.5856	0.5536	0.4949	0.5597	0.5932	0.5886	0.5752	0.5722	0.5728	0.5576	0.5743	0.5814
**C6**	0.5546	0.5408	0.5722	0.543	0.5432	0.4763	0.5772	0.5769	0.5537	0.5577	0.5572	0.5389	0.5618	0.5702
**C7**	0.5191	0.5183	0.55	0.5097	0.5192	0.5163	0.4832	0.5495	0.5329	0.5266	0.5323	0.5131	0.5262	0.5345
**C8**	0.5085	0.5118	0.5387	0.5058	0.5073	0.5077	0.544	0.4752	0.5167	0.5119	0.521	0.5141	0.5204	0.5285
**C9**	0.513	0.5183	0.5386	0.5009	0.5118	0.5057	0.5379	0.5381	0.4601	0.524	0.5235	0.5111	0.5254	0.5254
**C10**	0.5256	0.531	0.56	0.526	0.5209	0.5228	0.5633	0.5602	0.5414	0.4743	0.5394	0.519	0.5429	0.538
**C11**	0.5396	0.5421	0.5663	0.5332	0.5345	0.5238	0.5603	0.557	0.5445	0.5413	0.4813	0.5303	0.5504	0.5522
**C12**	0.5506	0.5379	0.573	0.5399	0.5409	0.5303	0.5737	0.5687	0.5527	0.5495	0.5537	0.4749	0.5569	0.5632
**C13**	0.5312	0.5229	0.5372	0.5084	0.5237	0.5129	0.5437	0.5452	0.5266	0.5293	0.5257	0.5148	0.4685	0.5371
**C14**	0.5419	0.5286	0.5611	0.5274	0.5323	0.5228	0.5605	0.5605	0.5419	0.5493	0.5486	0.5218	0.5383	0.484
